# New-York Journal of Medicine

**Published:** 1845-06-01

**Authors:** 


The New-York Journal of Medicine and the Collateral Sciences.—We perceive that this periodical, which was originated by the late, lamented Dr. Forry, is to be continued under the editorial management of Dr. C. A. Lee. From the evidences before the Medical Public of the erudition and industry of Dr. Lee, together with his ability as a writer, we entertain no fears that its reputation will deteriorate in his hands. We hope he will so identify himself with its character and success, that its permanency may be secured.

